# Comparison between direct and reverse electroporation of cells in situ: a simulation study

**DOI:** 10.14814/phy2.12673

**Published:** 2016-03-23

**Authors:** Leila Towhidi, Delaram Khodadadi, Nataly Maimari, Ryan M. Pedrigi, Henry Ip, Zoltan Kis, Brenda R. Kwak, Tatiana W. Petrova, Mauro Delorenzi, Rob Krams

**Affiliations:** ^1^ Department of BioengineeringImperial College LondonLondonUnited Kingdom; ^2^ Department of Pathology and ImmunologyUniversity of GenevaGenevaSwitzerland; ^3^ Department of Medical Specializations – CardiologyUniversity of GenevaGenevaSwitzerland; ^4^ Department of OncologyLausanne University Hospital and University of LausanneLausanneSwitzerland

**Keywords:** Electroporation, high‐throughput techniques, transfection efficiency

## Abstract

The discovery of the human genome has unveiled new fields of genomics, transcriptomics, and proteomics, which has produced paradigm shifts on how to study disease mechanisms, wherein a current central focus is the understanding of how gene signatures and gene networks interact within cells. These gene function studies require manipulating genes either through activation or inhibition, which can be achieved by temporarily permeabilizing the cell membrane through transfection to deliver cDNA or RNAi. An efficient transfection technique is electroporation, which applies an optimized electric pulse to permeabilize the cells of interest. When the molecules are applied on top of seeded cells, it is called “direct” transfection and when the nucleic acids are printed on the substrate and the cells are seeded on top of them, it is termed “reverse” transfection. Direct transfection has been successfully applied in previous studies, whereas reverse transfection has recently gained more attention in the context of high‐throughput experiments. Despite the emerging importance, studies comparing the efficiency of the two methods are lacking. In this study, a model for electroporation of cells in situ is developed to address this deficiency. The results indicate that reverse transfection is less efficient than direct transfection. However, the model also predicts that by increasing the concentration of deliverable molecules by a factor of 2 or increasing the applied voltage by 20%, reverse transfection can be approximately as efficient as direct transfection.

## Introduction

The discovery of the human genome has unveiled new fields of genomics, transcriptomics, and proteomics (Duscher et al. 2015; Stegle et al. 2015; Taher et al. 2015; Wes et al. 2016). This has produced a paradigm shift on understanding the pathogenesis of disease, which is now increasingly defined by gene signatures and gene networks (Duscher et al. 2015; Stegle et al. 2015; Taher et al. 2015; Wes et al. 2016). These gene networks often consist of more than 1000 genes with a huge amount of interactions between them. As an approach to understand the topology and complex interactions of these gene networks, one needs to be able to manipulate genes either through activation or inhibition. However, the cell membrane is fairly impermeable to external molecules, including DNA and RNA (Xiang and Chen 2000; Wu et al. 2002; Dorsett and Tuschi 2004). To overcome this permeability barrier, transfection methods such as lipofection, viral transduction, and electroporation have been developed (Ramamoorth and Narvekar 2015; Silva et al. 2015). Lipofection is a lipid‐mediated transfection method that delivers molecules to the cell by means of a liposome that easily merges with the cell membrane (Ramamoorth and Narvekar 2015; Silva et al. 2015). Despite numerous efforts, the efficiency of this technique to transfer molecules is low in primary mammalian cells (Ramamoorth and Narvekar 2015; Silva et al. 2015). Viral transduction involves the transfer of molecules encapsulated in viral vectors such as lentivirus or adenovirus. The limitations of viral transduction include labor insensitivity causing low throughput, need for safety measures, insertional mutagenesis, DNA package size limit, and immunogenicity (Ramamoorth and Narvekar 2015; Silva et al. 2015). For electroporation, an electric pulse is applied to the cell to permeabilize the cell membrane and allow uptake of external molecules. Under optimized pulse parameters, the membrane returns to its intact state (Neumann et al. 1982; Felgner et al. 1987; Plank et al. 2003; Bonetta 2005). Recent studies have shown that electroporation or nucleofection are indeed the most efficient transfection methods for difficult‐to‐transfect primary cell types (Gilbert et al. 2008; Lu et al. 2008).

In conventional electroporation, cells are first detached from the substrate and then exposed to electric pulses in the presence of the desired extracellular molecules which are dissolved in the cell suspension. The problem with this approach is that electroporation of cells in suspension can adversely affect cell viability and normal cellular function (Gowrishankar et al. 2006; Jain et al. 2009). Further, as the cells are floating, there is no spatial or temporal control over the area of electroporation, nor is there a mechanism to track individual electroporated cells. To overcome these limitations, several in situ approaches for adherent cells have been developed. In one study, a pulse was applied to cells seeded on a glass petri dish by placing two parallel wire electrodes at the sides of and in contact with the cell layer. In this case, the applied electric field is not uniform on all cells due to the shielding of neighboring cells. In another approach, one of the electrodes was made of conductive gold (Yamauchi et al. 2004), which is also used for seeding of the cells, and the electric pulse was applied by placing another electrode above the cells with pulsing buffer solution in between the electrode and the seeded cells. This provides a uniform electric field on the cells, but, as the gold substrate is not transparent, the setup is not compatible with microscopy imaging. In order to solve this problem, semiconductive indium tin oxide (ITO)‐coated glass slides were used instead of gold‐plated substrates. In addition to electric conductivity and biocompatibility, which renders ITO glass slides suitable for electroporation, optical transparency provides the capability to observe and examine the cells by microscope (Raptis and Firth 2008; Raptis et al. 2008; Santra et al. 2013).

There are two types of in situ electroporation techniques: direct and reverse. In the case of direct transfection, the nucleic acids are in the medium above the cells, and a pulse with appropriate parameters is applied to the adherent cells for transfection. For reverse transfection, the nucleic acids are first added to the substrate and then the cells are seeded on top of them. A pulse with optimized parameters is applied at the desired time to the cells seeded on the substrate covered by nucleic acids. Direct electroporation has been successfully applied in previous studies (Li and Ma 2001; Li 2004); on the other hand, reverse transfection has gained more attention recently. Reverse transfection requires fewer nucleic acids for experiments, so it is more cost‐effective, and it has the potential advantage of allowing high‐throughput experiments (Li and Ma 2001; Li 2004). The approach is to seed cells onto a surface that is typically coated with either siRNA or cDNA/CRISPr, which needs to be transfected into the cells by then applying an electric pulse (Li and Ma 2001; Li 2004). For a high‐throughput experimental design, different nucleic acids need to be dispensed at different spots over a suitable substrate to allow the cells attached to these spots to be efficiently reverse transfected without cross contamination. The conditions leading to optimal transfection efficiency are currently unknown and need to be further studied.

The aim of this article is to present a model that describes variations in cell membrane permeability and molecule uptake during electroporation as a function of electric pulse parameters and molecular concentrations. This model will aid the optimization of reverse transfection in high‐throughput microarray experiments by predicting necessary changes in concentration of molecules and parameters of electroporation to increase transfection efficiency of reverse electroporation, which will be compared to optimized protocols of direct electroporation reported previously.

## Methods

### Model development

**Figure 1:**
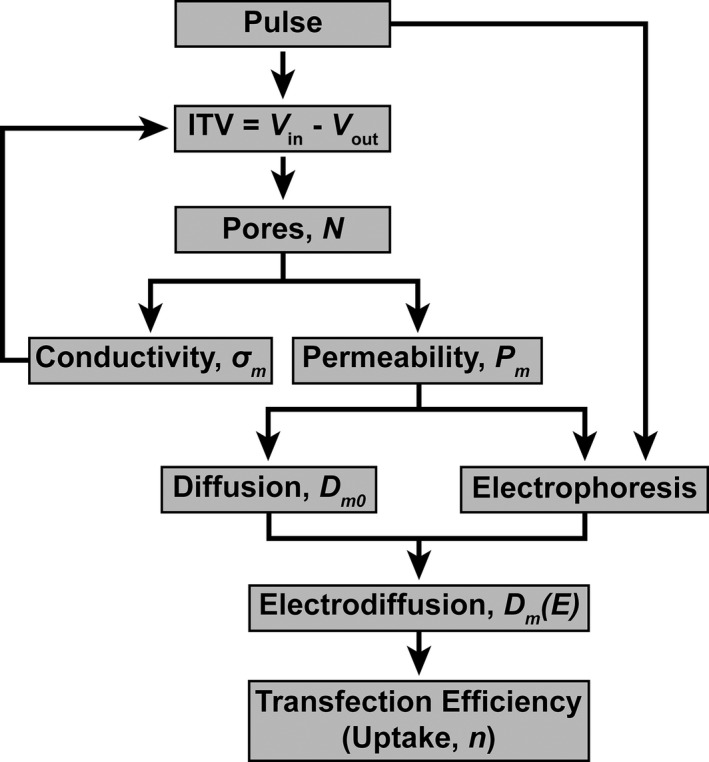
Electroporation and uptake model that is implemented in this article.

The present model is based on observations of endothelial cells, and it describes their behavior during electroporation. A flowchart describing the entire process is presented in Figure 1. Where possible, we have used analytical solutions to test the simulation. The factors that can affect the simulation results are the chosen module in the software, the number of meshes, and the specific solver for solving the problem in the software package. Besides, the constructed geometry could be important in the result. For example, in the analytical solution the electric field is considered to be uniform. In order to have a completely uniform electric field in the simulation, one has to consider electrodes with infinite size. This is practically impossible in the simulations. Therefore, we had to consider big enough electrodes so that the edge effects could be ignored and to meet the uniform electric field requirement. Therefore, a comparison between simulation results and the analytical solutions was essential. The rest of the model was made based on this initial module, solver, tolerance, and size.

As electroporation is strongly affected by cell shape, and analytical solutions do not exist for complex cell shapes, we subsequently developed numerical techniques to solve the equations for a single endothelial cell, a monolayer of endothelial cells, and a small cluster of cells. All simulations were performed with COMSOL version 4.4.

### Cell geometries

For benchmarking, a suspended spherical cell with a radius of 10 *μ*m and membrane thickness of 5 nm was considered between two parallel electrodes filled with a medium characteristic for the pulsing buffer. The nanometer range for the membrane thickness is extremely small compared with the bulk of the cell, and physically including this small thickness for the membrane in the geometry is not practical considering the inherent limit for mesh density in COMSOL. Hence, we decided to consider the cellular membrane as a boundary condition (Pucihar et al. 2006; Pucihar et al. 2009). The simulation result for this model was compared with the available analytical solution.

**Figure 2:**
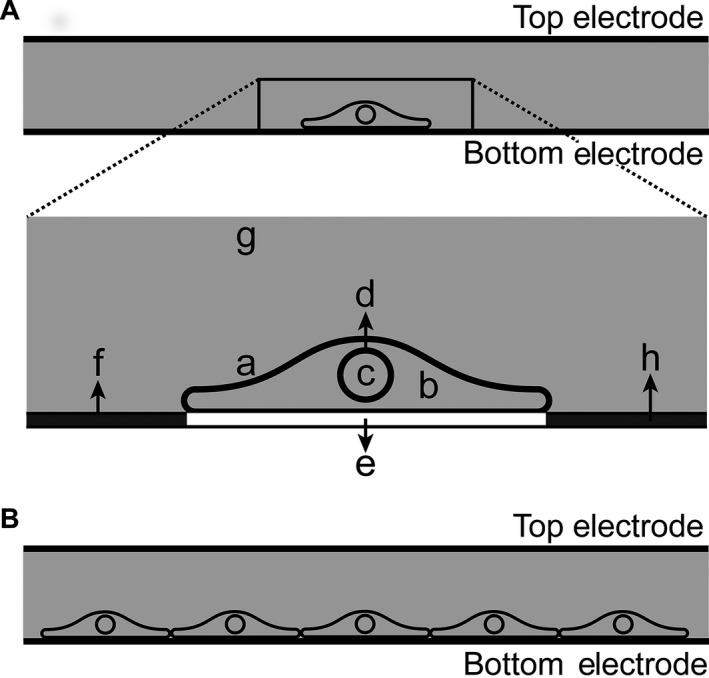
(A) Geometry of an attached cell used in the modeling, including cell membrane, a; cytoplasm, b; nucleus, c; nucleus membrane, d; material below the cell, e; material covering the slides around the cell, f; buffer on top of the cell, g; and bottom electrode, h. (B) Geometry of adjacent cells in a multicellular layer.

The geometry of the surface adherent endothelial cell was based on literature measurements, and each cell had a basal length of 58 *μ*m and maximum height of 10 *μ*m (Song et al. 2013). Furthermore, each endothelial cell included a nucleus with radius of 4 *μ*m and membrane thickness of 10 nm. Similar to the spherical cell model, membranes were modeled by assigning a boundary condition in the COMSOL modules. For this model –in consistence with previously published experimental methods in which an extracellular matrix was used for better cell attachment – the cells were positioned on a matrix layer (label E of Fig. 2A) and submerged in pulsing buffer (labels G and F of Fig. 2A).

Endothelial cells adhere easily to their substrates, where they form monolayers; therefore, a monolayer of 15 cells covering the entire bottom electrode was modeled. The electrodes were 80 *μ*m apart and 0.87 mm long. To reduce the computational time and memory requirements for solving the monolayer, the model was simplified to a single cell while the rest of cells were replaced with a layer of the same parameters as the cell membrane. The model is similar to Figure 2A, whereby F now contains information of the cell membrane. This assumption is validated later by comparing the simulation results of the simplified model with the real model considering all of the cells.

As explained for high‐throughput experiments using microarrays, cells attach to the immobilized spots of cDNA/siRNA. Therefore, when using these microarrays, cells do not cover the entire surface as a monolayer. Instead, they form a cluster or multicellular layer only at the location of the spot. To represent the multicellular layer, only five of the in situ cells were placed next to each other on the bottom electrode leaving the rest of the electrode empty (Fig. 1B).

### Meshing

For a spherical cell model, COMSOL predefined “normal” mesh using free triangular elements as sufficient for correct computation and ITV benchmarking. This mesh generates 994 elements and 2335 degrees of freedom.

For the surface adherent cell model, due to the irregularity of its geometry and the demand for correct computation of the electric field and the uptake model in the time‐dependent studies, a finer mesh was required. Construction of the mesh in all of the surface adherent models used a built‐in predefined “fine” mesh. Due to the fact that the surface adherent cell geometry was very small compared with the electrodes, a smaller mesh was required for the domain and boundary of the cell, especially the cell membrane where property changes were considerable and important. A very fine and extremely fine mesh was considered for the cell membrane and the curved areas.

### Exposing the cells to the electric field

During electroporation, when the cell is exposed to an appropriate short‐duration high‐voltage electric pulse, a voltage difference was induced in the cell bilayer membrane. For our simulations, the bottom electrode was set to ground (0 V), while the top electrode was set to a voltage of 1.36 V. These values were obtained from the relationship *V* = *E* * *d*, where *E* is the electric field that was desired (170 V cm^−1^) and *d* was the distance between the two electrodes, which was set to 80 *μ*m unless otherwise stated.

For static benchmarking and the static study of the attached cell, the relative permittivity was set to 5, and the electric conductivity was considered as a constant with a value of 5 × 10^−7^ S m^−1^. The material inside the cell was considered as porous cytoplasm with an electric conductivity of 0.3 S m^−1^ and relative permittivity of 80. The material in extracellular media was considered as pulsing buffer with properties expressed in Appendix 2 (Pucihar et al. 2006; Pucihar et al. 2009). Electric potential of intracellular and extracellular media was calculated using equation (A1).

Electric potential of intracellular and extracellular media was calculated using equation A1 in which the parameters *σ*_i_, *ε*_i_ and *σ*_e_, *ε*_e_, are the electric conductivity and relative permittivity of the material in each domain, respectively.

In order to couple the intracellular and extracellular media, a local flux normal to the surface was calculated (using eq. A2), and induced transmembrane voltage (ITV = *V*_in_ − *V*_out_) was determined from this equation as the potential difference between two sides of the membrane.

For modeling a spherical cell, to avoid time‐consuming simulation of a 3D geometry, we used a 2D axisymmetric study in COMSOL. A spherical cell with a radius of 15 *μ*m was considered between two electrodes 200 *μ*m apart. The simulation used the AC/DC module and the electric currents mode in COMSOL. Under the AC/DC module, boundary conditions were assigned to the membranes under “contact impedance” mode. The boundary condition parameters were a membrane thickness of 5 nm, relative permittivity of 5, and electric conductivity of 1 × 10^−4^ S m^−1^ (Pucihar et al. 2009; Rems et al. 2013). The material for cell cytoplasm was set to have an electric conductivity of 0.3 S m^−1^ and a relative permittivity of 80 (Pucihar et al. 2009). The simulation result for ITV in this case was compared with the analytical solution (ITV = $\frac{3}{2}$ *E R* cos(*θ*) ), where *E* is the static electric field magnitude, *R* is the cell radius, *θ* is the polar angle with respect to the field direction.

For a single‐attached cell, similarly, under the AC/DC module, boundary conditions were assigned to the membranes under contact impedance mode. The boundary condition parameters were a membrane thickness of 5 nm, relative permittivity of 5, nucleus membrane thickness of 1 nm, and electric conductivity of 1 × 10^−4^ S m^−1^ (Pucihar et al. 2009; Rems et al. 2013). The material for cell cytoplasm was set to have an electric conductivity of 0.3 S m^−1^ and a relative permittivity of 80 (Pucihar et al. 2009). The conductivity and relative permittivity inside the nucleus was considered as 0.5 S m^−1^ and 80, respectively (Rems et al. 2013).

For the static condition, a constant voltage was applied to the upper electrode, while in the dynamic condition, an electric pulse with a duration of 10 msec and rising and falling time of 2 *μ*sec was applied to the electrode.

The material used for the cell substrate and media (E and G in Fig. 2A) was different for direct and reverse electroporation. In direct transfection, cells were seeded on an extracellular matrix layer (E has parameters of extracellular matrix), and pulsing buffer containing siRNA was added on top of the cells (G has parameters of pulsing buffer containing siRNA). The properties for these materials are given in Appendix 2. In reverse transfection, cells were seeded on both siRNA and extracellular matrix, then pulsing buffer was inserted on top of the cell (E has parameters of mixture of extracellular matrix and siRNA, and G has parameters of pulsing buffer containing siRNA).

### Pore formation

When ITV increases after application of the pulse, it will cause structural perturbations to the cell membrane. As a consequence, pores start to develop in the membrane and it becomes partially permeable. This process was characterized by a partial differential equation (DeBruin and Krassowska 1998, 1999), as described in equation (A3) of Appendix 1, where N is the pore density induced in the membrane during the electric pulse, *N*_0_ is the initial equilibrium pore density in the nonelectroporated membrane, and parameters *q*,* α*, and *V*_ep_ describe the characteristics of the electroporation process (Pucihar et al. 2009; Rems et al. 2013). The numerical values of these parameters are presented in Appendix 2. In COMSOL, it is implemented through the PDE module under the “Weak Form Boundary PDE” interface that can solve the equation and calculate the pore density *N*(*t*).

Under the static condition, the membrane conductivity was considered constant and set at *σ*_mo_ = 5 × 10^−7^ S m^−1^, which is the measured natural conductivity of the cell membrane. However, during electroporation, an increase in the formation of pores resulted in an increase in the leakage of the membrane and consequently the conductivity of the cell membrane. Conductivity of pores is directly proportional to pore density, as is reflected in equation (A4) of Appendix 1, where *N*(*t*) is the pore density calculated previously by equation (A3), *r*_p_ and *σ*_p_ are the radius and internal conductivity of a single pore, respectively, and *d*_m_ is the cell membrane thickness. Numerical values for these parameters are given in Appendix 2.

The total membrane conductivity *σ*_m_ is then expressed as the sum of *σ*_m0_ and *σ*_ep_, where *σ*_m0_ is the passive membrane conductivity (5 × 10^−7^ S m^−1^) and *σ*_ep_ is the increase in conductivity due to electroporation (Rems et al. 2013). The AC/DC module of COMSOL incorporates this variable conductivity for the cell membrane conductivity (eq. A4), which is regularly updated based on the pore density value during electroporation. Therefore, during electroporation, with increasing pore density, the conductivity of each point on the cell membrane increases, which in turn affects ITV and which consequently affects the pore density again. Upon formation of pores in the membrane, permeability of the membrane increases as well.

### Permeability changes and transport through the membrane

During the pulse, cDNA or siRNA molecules transfer into the cell through the pores. The transport of the molecules through the membrane is an interactive transport via transient contacts of the molecule with the lipids of the pore edge (Neumann et al. 1998). The main mechanisms in this transport through the pores are diffusion and electrophoresis.

Diffusion obeys Fick's first law of diffusion, whereby the diffusion coefficient for the membrane is expressed as the product of membrane permeability (*P*_m_) and membrane thickness (*d*_m_) (Neumann et al. 1999). The second mechanism is electrophoresis, which is the movement of charged molecules due to the influence of an electric field. When a charged molecule with charge *q* is exposed to an electric field *E*, a force *F* is exerted on the molecule which is defined by Coulomb's law (Appendix 1). As a consequence, movement of negatively charged molecules such as cDNA or siRNA is further enhanced due to the polarity of the applied electric field. The combination of diffusion and electrophoresis is called electrodiffusion and is defined by equation (A7), where D_m0_ is the diffusion coefficient in the electroporated membrane obtained by equation (A7), *D*_m_(*E*) is the electrodiffusion coefficient in the electroporated membrane which is to be calculated, *Z*_eff_ is the effective charge number (with sign) of the transporter molecule (which is considered similar to DNA), *e*_0_ is the elementary charge, *k* is Boltzmann constant, and *T* is the temperature(Neumann et al. 1999). The numerical values for these parameters are given in Appendix 2.

The above discussion motivates expressing the permeability of a cell membrane as the sum of these two distinct mechanisms. The transport mechanisms of diffusion and electrophoresis of molecules is implemented using the Chemical Species Transport module in COMSOL. It considers the electrodiffusion coefficient, *D*_m_(*E*) to solve the Fick's law of diffusion. The initial concentration of siRNA molecules is set to 5 μmol L^−1^ for both direct and reverse electroporation.

The Chemical Species Transport module uses pore density *N*(*t*) obtained in the PDE module (in section 2.1.2) to calculate the permeability *P*_m_, then the diffusion coefficient , *D*_m0_ , and consequently the electrodiffusion coefficient, *D*_m_(*E*), which is inputted through “Thin diffusion barrier” for the membrane. For simplicity, “Thin impermeable barrier” was assigned to the nucleus membrane. This module can also account for the poroelasticity of the cytoplasm where the porosity was set at 0.75 (Moeendarbary et al. 2013).

### Cellular uptake

After the molecules move into the cell, transfection efficiency can be estimated based on the total number of molecules that have moved inside the cell, referred to as the cell “uptake.” The uptake is denoted by *n* and can be computed by integrating the number of molecules that have transported through the cell membrane over time and cell surface, according to equation (A8) in Appendix 1, where j is the total flux, S is the surface of the cell membrane, τ is the time at which that uptake is to be calculated, and *N*_A_ is Avogadro's number given in Appendix 2 (Towhidi and Miklavcic 2010).

## Results

### Benchmarking

**Figure 3:**
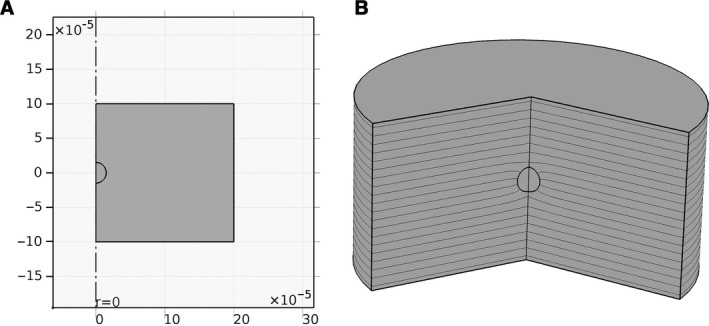
(A) 2D axisymmetry model for a single spherical cell between two plate electrodes. The dashed‐dot line shows the symmetry axis. The upper and lower electrodes are shown by arrows in the figure. (B) A section of the 3D view of the model. The lines in the figure show the contours of electric potential. It can be seen that the lines are bent around the cell due to the presence of the cell.

We tested the implementation of the numerical software with a suspended spherical cell in static condition, as an analytical solution was present in the literature. A constant uniform electric field of 170 V cm^−1^ was applied to the cell by the two electrodes (as shown in Fig. 3A, B). In our model, with the chosen simulation solver parameters, meshes, and geometry, the largest difference between these two results occurs at the maximum of ITV (data not shown) and it is less than 1%, and therefore, the results have more than 99% consistency, proving the accuracy of our numerical approach.

### Comparison between static and dynamic study for a single attached cell

**Figure 4:**
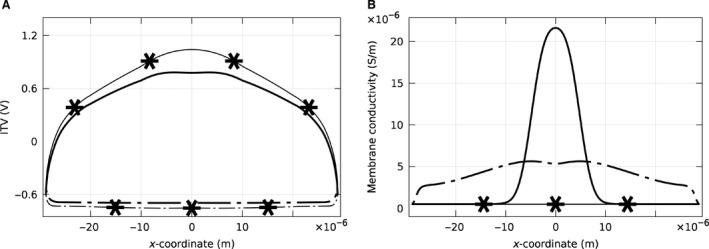
(A) Induced transmembrane voltage and (B) membrane conductivity for a single cell attached to a conductive slide. The applied electric field was 400 V m^−1^. The lines with the asterisks belong to the static case in which conductivity of the membrane is considered constant. The lines without asterisks correspond to the dynamic case in which conductivity of the membrane changes as a function of the ITV. The solid lines demonstrate the results for the apical side, while the dash‐dot lines show the results for the basal side of the cell. The ITV in the static case for a single cell is larger on the apical side. This results in a larger conductivity in the dynamic case, which, in turn, causes the reduction of ITV so that the ITV has almost the same maximum value for both the apical and basal sides.

The effect of a pulse on ITV of the apical and basal parts of the membrane of a single attached cell in static and dynamic cases was first considered. The cell was modeled on top of an extracellular matrix layer, surrounded by pulsing buffer (Fig. 2A). A high electric field (400 V m^−1^) was chosen to facilitate comparison of the static and dynamic cases. In the static case, the apical ITV along the cell membrane (the solid line with asterisk in Fig. 4A) was nonuniform and clearly larger than the ITV in the basal part of the membrane (dashed line with asterisk in Fig. 4A). The nonuniform ITV along the cell membrane was due to the voltage drop through the area adjacent to the cell (conductive slide covered by pulsing buffer). In the dynamic case, the conductivity of the membrane was not constant and depended on the magnitude of the ITV on the membrane. As shown previously (Fig. 4A), the ITV is nonuniform along the cell membrane. A larger ITV resulted in more pores on the membrane and a consequent increase in the conductivity (Fig. 4B). As the membrane acts like a capacitor that can be charged with a specific time constant, these variations were dependant on time and the pulse parameters. The change in the conductivity on the apical and basal part of the membrane was different. In turn, the dotted line with asterisks shows membrane conductivity in the static case. Further, the increase in the conductivity of the membrane in the dynamic case, in turn, resulted in a voltage drop on the membrane (Fig. 4A, solid and dashed line for apical and basal).

### ITV, pore density, and permeability for the monolayer model

**Figure 5:**
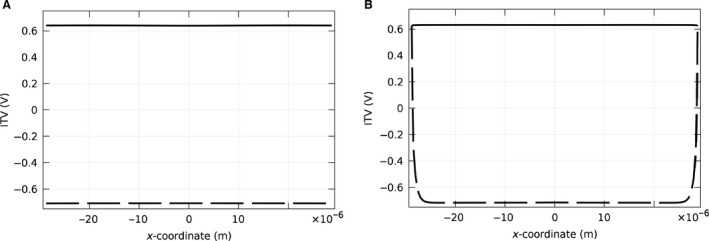
ITV in static study for a single cell in (A) a real monolayer of the cells on the substrate and (B) a simplified model replacing all other cells with a layer that has characteristics of the cell membrane. The solid lines show the ITV for the apical side, and the dashed lines indicate ITV for the basal side of the cell. These results show that the actual model of a monolayer and the simplified model are within 95% of each other.

As endothelial cells usually form a monolayer, we next modeled a monolayer of 15 cells covering the entire electrode. The applied electric field was 170 V cm^−1^. The results demonstrated that the spatial distribution of ITV was uniform over all 15 cells, and the ITV was slightly larger on the basal side of the cell monolayer (data not shown). Because the monolayer simulations require extensive computational time and memory, we simplified the monolayer model by replacing the cells with a homogeneous layer containing the same parameters as a single‐cell membrane (Fig. 2A). To validate this simplified model, we compared the ITV result from this model to the actual model of 15 adjacent cells and found them to be within 95% of each other (Fig. 5). Thus, all additional results were obtained using the simplified monolayer.

**Figure 6:**
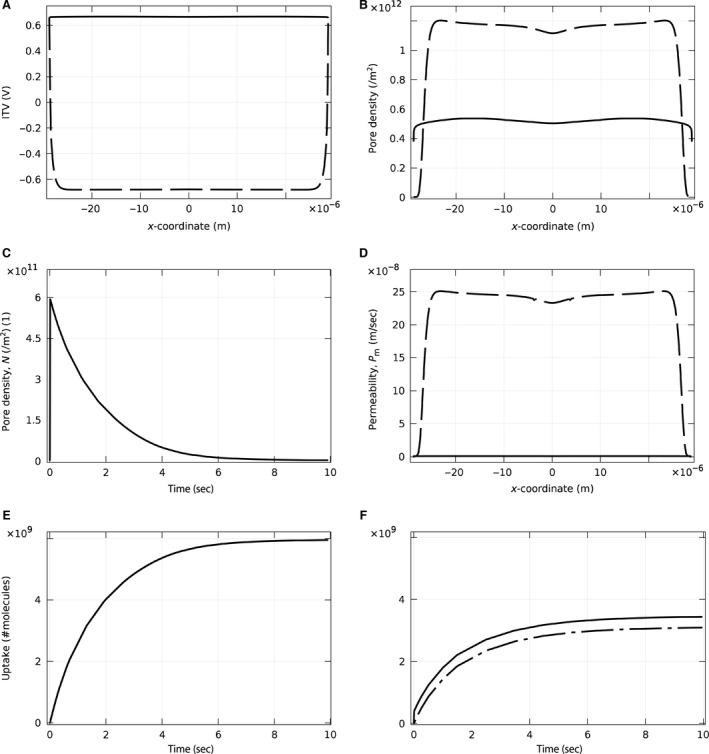
Study of the dynamic case for electroporation of endothelial cells within a monolayer, using a 10 msec pulse of 150 V m^−1^. (A) Induced transmembrane voltage on the apical (solid line) and on the basal (dashed line) side of the membrane. The ITV is almost uniform along the membrane and slightly larger for the basal side compared with the apical (0.68 on basal and 0.67 on apical). (B) Resulting pore density on the apical (solid line) and basal (dashed line) sides of the membrane. Due to the larger ITV on the basal side during the pulse, the pore density is larger. The permeability related to diffusion has the same trend (not shown). (C) Shows the evolution of pore density at the highest point of the cell with time after the pulse ends. The pores reseal after approximately 10 sec, while ITV vanishes immediately after the pulse ends. (D) Permeability related to electrodiffusion for reverse electroporation at the apical (solid line) and the basal (dashed line) sides of the cell. The permeability of the basal membrane gets even larger for reverse electroporation. (E) and (F) show the uptake of the cell in direct and reverse electroporation, respectively. It is clear that direct electroporation is more effective. In (F), the dot‐dash line shows the uptake just due to the diffusion, and the solid line shows the uptake due to the electrodiffusion. Although the main factor of the uptake is diffusion, electrodiffusion has significant effect on the uptake in reverse electroporation.

Next, the (simplified) monolayer was modeled as seeded on extracellular matrix (label E of Fig. 2A) and immersed in pulsing buffer containing siRNA (label G of Fig. 2A). For the dynamic case of direct electroporation, temporal changes in conductivity of the membrane were considered, and consequently, ITV at different points of the membrane was subjected to changes over time. Shortly after the pulse started, conductivity and ITV reached equilibrium so that the ITV at the end of the pulse demonstrated a nearly constant profile on both sides of the cell layer (Fig. 6A). Based on the geometry of this case and the pulse parameters used, the equilibrium value for the basal side was slightly larger than the apical side (0.67 on apical and 0.68 on basal). The equilibrium value can exhibit even larger differences between apical and basal sides for lower voltages. As a result of this larger value of ITV, pore density was larger on the apical side compared with the basal side (Fig. 6B). By the end of the pulse at 10 msec, pore density had increased significantly. Thereafter, although the ITV had vanished, pore density exhibited an exponential decrease over time. We found that the time of complete reseal depended on the initial pulse parameters. In this case, at the highest point in the cell, the pores were nearly resealed after 10 sec (Fig. 6C), which is consistent with the experimental results (Sukharev et al. 1992). Interestingly, the pore density on the basal side of the middle of the cell layer had a slight drop due to a slightly larger ITV locally (Fig. 6B). This larger ITV caused a slightly larger pore density (based on eq. A3) and hence an increase in conductivity (based on eq. A4), which, in turn, reduced ITV and resulted in a reduction of the pore density locally. Finally, the permeability of the cell layer along the membrane was calculated. As the permeability due to diffusion is proportional to pore density (see Appendix 2), the behavior of these two quantities were similar (data not shown).

Using these same modeling parameters, reverse electroporation demonstrated similar results to the direct electroporation results described above (data not shown). However, electrophoresis is an extra effect that can change permeability of the membrane. This effect is only operative whenever the force due to the pulse applied to the charged molecules is toward the intracellular media. In the experimental setup, the lower electrode was connected to the ground, so the force on the siRNA would be upwards. Hence, the electrophoresis would be effective only in reverse electroporation (Fig. 6D). Note that the permeability in the basal part of the cell increased considerably due to this mechanism.

### ITV, pore density, and permeability for the multicell model

**Figure 7:**
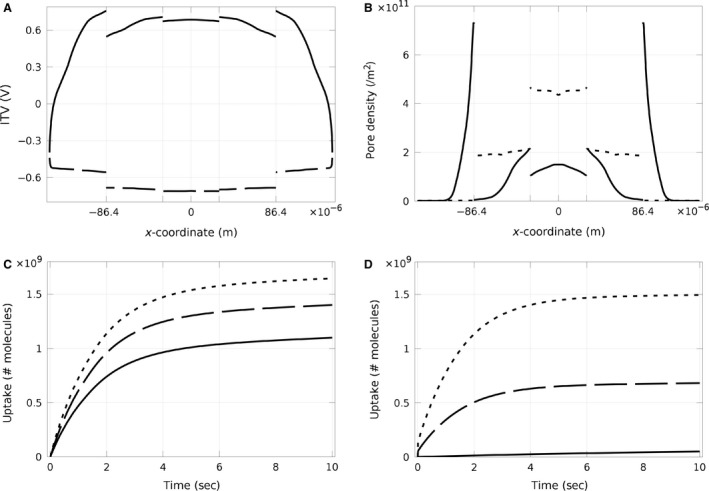
Dynamic case study for electroporation of five adjacent endothelial cells in a multicellular layer or cluster. (A) Induced transmembrane voltage and (B) pore density of a 10 msec pulse of 150 V m^−1^ at the apical (solid line) and the basal (dashed line) side of five adjacent cells on a spot. The vertical grids in (A) and (B) show the location of each cell, considering the middle cell on *x* = 0. The ITV and consequently pore density are not uniform along the membrane of each cell and very different on the adjacent cells. (C) and (D) display the uptake of different cells in the multicellular model for direct and reverse electroporation, respectively. The solid lines, dashed lines, and dotted lines are related to the cells on the edges, cells adjacent to the edge cells, and cells adjacent to the middle cell, respectively. It is clear that uptake of the cell in the middle is the highest, and uptake for the cells on the edges is the lowest.

In high‐throughput experiments using microarrays, cells are seeded on immobilized spots of cDNA/siRNA. Unlike a monolayer covering the entire substrate, cells seeded onto these spots only cover a local area of the substrate. We call these local regions of cells a multicellular layer or cell clusters. Therefore, a multicellular layer was modeled consisting of five cells covering a section of the electrode (Fig. 2B) and the electric pulse was applied in a similar manner as detailed above. In comparison to the monolayer discussed above, ITV was less uniform over the multicellular layer due to the voltage drop at the edges of the individual cells (Fig. 7A). This voltage drop was observed in the results for an individual cell, but now these drops occurred at the edge of the cell cluster. Correspondingly, pore density on the apical side was highest on the edge of the cells, but only on the side of the cell attached to the neighboring cell (Fig. 7B). In contrast, on the basal side, pore density was highest on the cell in the center. Regarding the cells in the middle of the layer, the basal side had a larger ITV compared to their apical sides, which caused a higher number of pores on the basal side of the cells in this region. Interestingly, the cells in‐between the edge and middle cells exhibited a drop in pore density on the basal side. This phenomenon is due to the increase in conductivity during electroporation, which locally decreases ITV and, in turn, pore density. In contrast, the basal pore densities for the cells at the edges were very low and the apical side of these cells showed high pore density only on the edges of the spot, whereas pore density on the other side of the cell was low. Finally, the time‐dependant behavior of pores resealing was similar between the multicellular layer and monolayer models.

It is to be noted that in our model, we considered only five cells and showed pore density varies along the cells (especially on the apical side of the edge cells) based on their positions. Although, in a typical experiment, there are usually more than 20 cells along the diameter of a cluster, meaning there would be a lower overall percentage of edge cells that would experience significantly reduced pore formation and more cells in the middle of the cluster that would experience a more uniform pore density; thus, the variations over the actual cell cluster as a whole would be lesser (almost similar to the monolayer case).

### “Direct” versus “Reverse” electroporation efficiency

The ultimate aim of this study was to compare direct versus reverse electroporation of cells in situ. siRNA uptake was computed for each cell in the monolayer model over time for both direct (Fig. 6E) and reverse (6F) electroporation. The multicellular layer model was also studied, and the uptake for direct (Fig. 7C) and reverse (Fig. 7D) electroporation was compared. Direct electroporation demonstrated an increase in the uptake of each cell of both models over time during the 10‐sec study (this computational time of the study was determined by the fact that pores mostly reseal after this time, as shown in Fig. 6C). Using the same pulse parameters and siRNA concentrations, reverse electroporation also showed an increase in uptake over time, but with only ~half of the final uptake of direct electroporation. This higher efficiency of direct electroporation occurred despite the fact that permeability was higher for reverse electroporation. There are two reasons for this result. First, the volume of siRNA below the cell in reverse electroporation is much less than above the cell in direct electroporation. Second, it was shown that after immobilization, less than 40% of the siRNA released due to applying a pulse (Yamauchi et al. 2004). Therefore, a lower concentration of siRNA (C/3) was considered as free siRNA in the model for reverse electroporation. As can be seen in Figure 6F, in reverse electroporation, the electrophoresis is considerably contributing to the uptake.

The multicellular model also showed that for both direct and reverse electroporation, the cells on the middle of the spot had the highest uptake and the cells at the edges had the lowest uptake. Uptake by the peripheral cells in the reverse electroporation case was particularly low, near zero. Despite variations in pore density along the cells in different positions, in both direct and reverse electroporation, the uptake of the middle cell is the highest and the uptake of the cells on the edge of the clusters is the lowest.

However, as mentioned before, we are interested in reverse transfection with application to high‐throughput experiments. Hence, we performed simulations to optimize the efficiency of reverse transfection (data not shown). The study indicated that efficiency of electroporation can be increased by increasing siRNA concentration and electric field strength, although the degree of such increases will still depend on the location of the cell on the spot. The uptake of the cell in the middle of the spot can improve with an increase in siRNA concentration of 1.5‐ to 2‐fold, but the cells on the edges will still not be able to reach the direct electroporation uptake with this change in concentration. Voltage had an even larger effect. Just a 20% increase in voltage leads to roughly the same uptake for the middle cell. But again, higher voltages are needed to have the same uptake for the cells on the edges. Maximizing both of these parameters, siRNA concentration and voltage, did lead to reverse transfection efficiencies that were comparable to those of direct transfection.

## Discussion

Previous experimental and modeling studies have examined the relationship between electroporation parameters such as voltage and species concentration versus efficiency of molecule uptake within the cell (Wilson et al. 1991). However, all of these studies assumed the cell geometry to be perfectly spherical, and no study has investigated the electroporation uptake efficiency of attached cells or compared the efficiency of direct versus reverse electroporation. In this study, we sought to develop a multiphysics model of electroporation that numerically calculates the ITV, pore density, membrane conductivity, membrane permeability, and species uptake by the cell to estimate the efficiency of direct versus reverse electroporation and create a numerical technique for optimizing the value of each of these parameters. The model employs a simplified 2D geometry (saving computational time and memory compared to 3D) that is adaptable to any cell geometry.

In this study, we modeled the geometry of endothelial cells (based on Yamada et al. [Yamada et al. 2010]) and considered several conditions. For electroporation of a single attached cell, we found a larger and more nonuniform ITV along the apical cell membrane due to the voltage drop from the area around the cell as it is a bare electrode covered with pulsing buffer. In contrast, the (simplified) cell monolayer model demonstrated a uniform ITV along the cell membranes because there is no voltage drop through the pulsing buffer as the whole slide is covered with cells, which translates to a uniform efficiency of electroporation across each cell membrane and over all cells of the monolayer. In the dynamic case for this cell monolayer, the conductivity of the membrane is a function of the ITV which results in a variable conductivity and time evolution of ITV. The final ITV at the end of the pulse on the basal and apical sides was nearly identical (but not exactly the same) (Fig. 6A). Thus, it can be inferred from these results that the uniformity of ITV within an electroporated cell layer increases with increasing number of cells in the layer. ITV uniformity is important because it is directly related to pore formation rate and corresponding molecule uptake (or permeability). In our model, pores start to form in the membrane directly after the pulse onset. The corresponding permeability at the start of the pulse was small, but it increased over the pulse duration, and although the ITV reaches zero immediately after the pulse ends, the pores reseal slowly and the membrane stays permeable for almost 10 sec. This important result confirmed that cells can still uptake molecules for a significant period after the pulse ends (Ryttsen et al. 2000). Finally, molecule uptake in direct versus reverse electroporation was considered. Since electrodiffusion is effective only in reverse electroporation, there is more pronounced uptake in the reverse approach for charged molecules (Fig. 6F). Despite this point, simulations show that the uptake was smaller (i.e., less efficient) for reverse electroporation (Fig. 6E, F). This result is due to the lower number of molecules in reverse versus direct electroporation with the same molecule concentration, wherein the molecules in the reverse case only come from the substrate, whereas in the direct case, the molecules are obtained from the entire volume of pulsing buffer above the cells. In addition, only 40% of the siRNA molecules bound to the substrate get released during a pulse, further lowering the total number of molecules available for uptake by the cells. To increase uptake efficiency of reverse electroporation in a multicellular layer, the siRNA concentration or the applied voltage should be also increased.

Reverse electroporation has drawn considerable attention recently, as it can be used for high‐throughput experiments that seek to assay genes on a large scale using cDNA or RNAi microarrays, which saves both time and cost (Jain et al. 2009; Kis et al. 2014). Experimental direct electroporation has been optimized previously. However, one complication with microarrays is that cells are seeded onto immobilized spots, so instead of a monolayer across the entire substrate, they only form clusters at the location of the individual spot. As can be inferred above from the comparison of ITV within a single cell versus a whole monolayer, our model predicted that clustering of cells into small groups leads to a less uniform ITV along the membrane of each individual cell, particularly for the cells at the edges (Fig. 7A). As a result, pore formation and permeability are not uniform across the cell cluster, wherein uptake is highest for the cells in the middle of the cluster and lowest for the cells on the edge of the cluster. Although nonuniform pore formation in clusters occurs in both direct and reverse electroporation, it is exacerbated in reverse electroporation (Fig. 7B). Thus, only the uptake of the middle cells in the cluster will be comparable in the optimized reverse electroporation protocol compared to that for direct electroporation, whereas the cells at the edge will be far less. This nonuniform uptake is a drawback of electroporation of cell clusters in this platform, in general, and it makes optimization of reverse electroporation to increase uptake efficiency difficult. Although the uptake is lower for cells at the cluster edge, our model only considered five cells in the cluster, whereas a typical experiment would include more than 20 cells per cluster. Therefore, edge cells experiencing reduced uptake would represent an overall much lower percentage of the entire cell population, resulting in an overall more uniform uptake (closer to that of the monolayer case). Nevertheless, we sought herein to use our model to additionally optimize two primary parameters of reverse electroporation to obtain a similar transfection efficiency as direct electroporation (which has been optimized previously), applied voltage, and siRNA concentration. We found that a roughly 20% increase in the applied voltage or a twofold increase in the siRNA concentration caused the uptake in reverse electroporation to improve to the level of direct electroporation for the middle cells of the cluster.

In summary, we developed a model to evaluate the transfection efficiency of reverse versus direct electroporation and optimize the parameters for reverse transfection in a microarray platform. We found that although adherent cells demonstrate a higher permeability through their basal side, transfection is more efficient through their apical side due to the larger number of molecules in the pulsing buffer volume above the cell versus those in the attachment area below the cell. In addition, just 40% of the siRNA molecules underneath the cell are released with the applied pulse. Therefore, reverse transfection is less efficient when using the same electroporation parameters. To obtain the same transfection efficiency between the two approaches for the microarray platform, our model predicted the need for either a twofold increase in siRNA concentration or a 20% increase in voltage in the reverse electroporation case compared with direct electroporation.

One limitation of this study is the use of a constant pore size. Although it is possible to incorporate varying pore radius into our model, the pore size was assumed to be constant similar to previous studies (DeBruin and Krassowska 1999; Gowrishankar et al. 2006). However, these studies have demonstrated that incorporating a variable pore size into the model does not have a significant effect on the results. For example, one study showed that increasing cell membrane conductance comes mainly from large pores (Krassowska and Filev 2007). This result means that until nascent (small) pores obtain a stable (large) pore size during their evolution, they have only minor effect on the cell membrane conductance. Moreover, it has been found that the resealing is primarily based on closing pores while the decrease in diameter contributes only to a minor extent, which implies that pore resealing is approximately an all‐or‐none process (Schwister and Deuticke 1985).

Although we focused on siRNA concentration and voltage herein, future work may assess other parameters such as pulse duration and the number of pulses. In addition, the strong dependency of the results on cell geometry and type motivates evaluation of real 3D cell geometries from imaging within multiple cell types, the optimized parameters of which could then be validated experimentally.

## Conflict of Interest

None declared.

## Appendix 1 Model Equations

1. (A1) $$-\nabla \left(\sigma_{i}\nabla V\right)-\nabla \frac{\partial \left(\varepsilon_{i}\nabla V\right)}{\partial t}=0.$$
2. (A2) $$n\cdot J=\frac{\sigma_{m}}{d_{m}}\left(V_{\mathrm{in}}-V_{\mathrm{out}}\right)+\frac{\varepsilon_{m}}{d_{m}}\left(\frac{\partial V_{\mathrm{in}}}{\partial t}-\frac{\partial V_{\mathrm{out}}}{\partial t}\right)$$
3. (A3) $$\frac{dN(t)}{dt}=\alpha e^{{\left(\frac{\mathrm{ITV}}{V_{\mathrm{ep}}}\right)}^{2}}\left(1-\frac{N(t)}{N_{0}}e^{-q{\left(\frac{\mathrm{ITV}}{V_{\mathrm{ep}}}\right)}^{2}}\right)$$
4. (A4) $$\sigma_{\mathrm{ep}}\left(t\right)=N\left(t\right)\frac{2\pi r_{p}^{2}\sigma_{p}d_{m}}{\pi r_{p}+2d_{m}}$$
5. (A5) $$P_{m}\left(t\right)=\left(N\left(t\right)\cdot \frac{D_{p}}{d_{m}}\right)*\pi r_{p}^{2}$$
6. (A6) $$D_{m0}=P_{m}\cdot d_{m}$$
7. (A7) $$D_{m}\left(E\right)=D_{m0}\left(1+\frac{Z_{\mathrm{eff}}\;e_{0}\;ITV}{kT}\right)$$
8. (A8) $$n=N_{A}\int_{t=0}^{\tau }\int_{s}j\;dS\;dt$$

## Appendix 2 Model Parameters

**Table:**

Parameter	Symbol	Value	References
Pore creation rate constant	*q*	2.46	(Pucihar et al. 2009; Rems et al. 2013)
Creation rate coefficient	*α*	1 × 10^9^ m^−2^S^−1^	(Pucihar et al. 2009; Rems et al. 2013)
Characteristic voltage of electroporation	*V*_ep_	170 mV	(Pucihar et al. 2009; Rems et al. 2013)
Equilibrium pore density	*N*_0_	1.5 × 10^9^ m^−2^	(Pucihar et al. 2009; Rems et al. 2013)
Pore radius	*r*_p_	0.76 × 10^−9^ m	(Rems et al. 2013)
Pore conductivity	*σ*_p_	0.0745 S m^−1^	(Rems et al. 2013)
Free diffusion coefficient	*D*_0_	5 × 10^−10^ m^−2^sec^−1^	(Towhidi and Miklavcic 2010)
Diffusion coefficients for the interactive transport	*D*_p_	*D*_0_/5	(Towhidi and Miklavcic 2010)
Effective charge number (with sign) for DNA	*Z*_eff_	−1	(Neumann et al. 1999)
Elementary charge	*e*_0_	1.60 × 10^−19^ C	(Neumann et al. 1999)
Boltzmann constant	*k*	1.38 × 10^−23^ JK^−1^	(Neumann et al. 1999)
Temperature	*T*	298 K	(Neumann et al. 1999)
Avogadro's number	*N*_A_	6.022 × 10^23^ mol^−1^	(Towhidi and Miklavcic 2010)
Circular cell radius	*R*	10 × 10^−6^ m	(Kotnik et al. 1997)
Cell membrane thickness	*d*_m_	5 × 10^−9^ m	(Pucihar et al. 2009; Towhidi and Miklavcic 2010; Rems et al. 2013)
Cell membrane electric conductivity (passive)	*σ*_m0_	5 × 10^−7^ S m^−1^	(Pucihar et al. 2009; Towhidi and Miklavcic 2010; Rems et al. 2013)
Cell membrane relative permittivity	*ε*_m_	5	(Pucihar et al. 2009)
Cytoplasmic electric conductivity		0.3 S m^−1^	(Pucihar et al. 2009; Towhidi and Miklavcic 2010; Rems et al. 2013)
Cytoplasmic relative permittivity		80	(Pucihar et al. 2009)
Nucleus radius		4 × 10^−6^ m	(Rems et al. 2013)
Nucleus membrane thickness		10 × 10^−9^ m	(Rems et al. 2013)
Nucleus membrane electric conductivity		1 × 10^−4^ S m^−1^	(Rems et al. 2013)
Nucleus membrane relative permittivity		7	(Rems et al. 2013)
Nucleus electric conductivity		0.5 S m^−1^	(Rems et al. 2013)
Nucleus relative permittivity		80	(Rems et al. 2013)
Extracellular matrix electric conductivity		0.01 S m^−1^	
Extracellular matrix relative permittivity		0.5	
siRNA + extracellular matrix electric conductivity		3.25 S m^−1^	
siRNA + extracellular matrix relative permittivity		80	
Pulsing buffer electric conductivity		1.8 S m^−1^	
Pulsing buffer relative permittivity		40	
siRNA + pulsing buffer electric conductivity		0.3 S m^−1^	
